# A Subpopulation of Foxj1-Expressing, Nonmyelinating Schwann Cells of the Peripheral Nervous System Contribute to Schwann Cell Remyelination in the Central Nervous System

**DOI:** 10.1523/JNEUROSCI.0585-18.2018

**Published:** 2018-10-24

**Authors:** Dan Ma, Bowei Wang, Malgorzata Zawadzka, Ginez Gonzalez, Zhaozong Wu, Bin Yu, Emma L. Rawlins, Robin J.M. Franklin, Chao Zhao

**Affiliations:** ^1^Wellcome Trust-Medical Research Council Cambridge Stem Cell Institute and Department of Clinical Neurosciences, University of Cambridge, Cambridge CB2 0AH, United Kingdom,; ^2^The Wellcome Trust/Cancer Research UK Gurdon Institute, University of Cambridge, Cambridge CB2 1QN, United Kingdom,; ^3^Department of Orthopaedic Surgery, Southern Medical University, Baiyun, Guangzhou 510515, P.R. China, and; ^4^Laboratory of Neuromuscular Plasticity, Nencki Institute of Experimental Biology, Pasteur 3, 02-093 Warsaw, Poland

**Keywords:** CNS remyelination, Foxj1, peripheral nerve, Schwann cells

## Abstract

New myelin sheaths can be restored to demyelinated axons in a spontaneous regenerative process called remyelination. In general, new myelin sheaths are made by oligodendrocytes newly generated from a widespread population of adult CNS progenitors called oligodendrocyte progenitor cells (OPCs). New myelin in CNS remyelination in both experimental models and clinical diseases can also be generated by Schwann cells (SCs), the myelin-forming cells of the PNS. Fate-mapping studies have shown that SCs contributing to remyelination in the CNS are often derived from OPCs and appear not to be derived from myelinating SCs from the PNS. In this study, we address whether CNS remyelinating SCs can also be generated from PNS-derived cells other than myelinating SCs. Using a genetic fate-mapping approach, we have found that a subpopulation of nonmyelinating SCs identified by the expression of the transcription factor Foxj1 also contribute to CNS SC remyelination, as well as to remyelination in the PNS. We also find that the ependymal cells lining the central canal of the spinal cord, which also express Foxj1, do not generate cells that contribute to CNS remyelination. These findings therefore identify a previously unrecognized population of PNS glia that can participate in the regeneration of new myelin sheaths following CNS demyelination.

**SIGNIFICANCE STATEMENT** Remyelination failure in chronic demyelinating diseases such as multiple sclerosis drives the current quest for developing means by which remyelination in CNS can be enhanced therapeutically. Critical to this endeavor is the need to understand the mechanisms of remyelination, including the nature and identity of the cells capable of generating new myelin sheath-forming cells. Here, we report a previously unrecognized subpopulation of nonmyelinating Schwann cells (SCs) in the PNS, identified by the expression of the transcription factor Foxj1, which can give rise to SCs that are capable of remyelinating both PNS and CNS axons. These cells therefore represent a new cellular target for myelin regenerative strategies for the treatment of CNS disorders characterized by persistent demyelination.

## Introduction

Remyelination is the process by which new myelin sheaths are restored to demyelinated axons (Franklin and ffrench-Constant, 2017). In the CNS, remyelination is mediated by oligodendrocytes newly generated from a widespread and abundant population of adult multipotent progenitor cells commonly called oligodendrocyte progenitor cells (OPCs) (Franklin and ffrench-Constant, 2017). Somewhat counterintuitively, demyelinated axons in the CNS can also be remyelinated by Schwann cells (SCs), the myelinating cells of the PNS (Blakemore, 1975). This atypical SC remyelination of the CNS occurs not only in experimental models of CNS demyelination, but also in a number of naturally occurring demyelinating pathologies of autoimmune, traumatic, genetic, and toxic origin (Ghatak et al., 1973; Itoyama et al., 1983, 1985; Duncan and Hoffman, 1997). It was originally believed that these CNS SCs were entirely of peripheral origin, immigrating into the damaged CNS following a breach in the astrocytic glia limitans (Franklin and Blakemore, 1993; Duncan and Hoffman, 1997). However, more recent genetic fate-mapping studies in which reporter proteins are expressed exclusively with adult OPCs have revealed that most are derived from OPCs and that very few are derived from myelinating SCs of the PNS (Zawadzka et al., 2010; Assinck et al., 2017). Nevertheless, these studies do not exclude the possibility that PNS cells other than myelinating SCs may provide a source of CNS SCs (Zujovic et al., 2010). Even though the adult PNS does not contain a population of progenitor cells equivalent to OPCs and PNS remyelination is often by mature SCs undergoing “dedifferentiation” into an immature progenitor state and generate remyelinating SCs (Jessen and Mirsky, 2005, 2016), there are other cells types that may potentially be sources of CNS SCs, especially those of neural crest origin such as nonmyelinating SCs (Remak cells) and endoneurial fibroblasts.

In the present study, we used an inducible Cre-Lox fate-mapping strategy to assess the contribution of Foxj1-expressing cells to the remyelination following lysolecithin-induced demyelination in the spinal cord. The original rationale for our choice of Foxj1-Cre was to establish whether Foxj1-expressing ependymal cells contribute to CNS remyelination given recent reports of their plasticity following traumatic spinal cord injury (Meletis et al., 2008). Although we found no evidence of Foxj1^+^ ependymal cells contributing to the generation of new remyelinating cells, we instead found expression of Foxj1 in nonmyelinating SCs and evidence that these cells are a source of CNS remyelinating SCs.

## Materials and Methods

### 

#### 

##### Animals.

**Figure 1.**
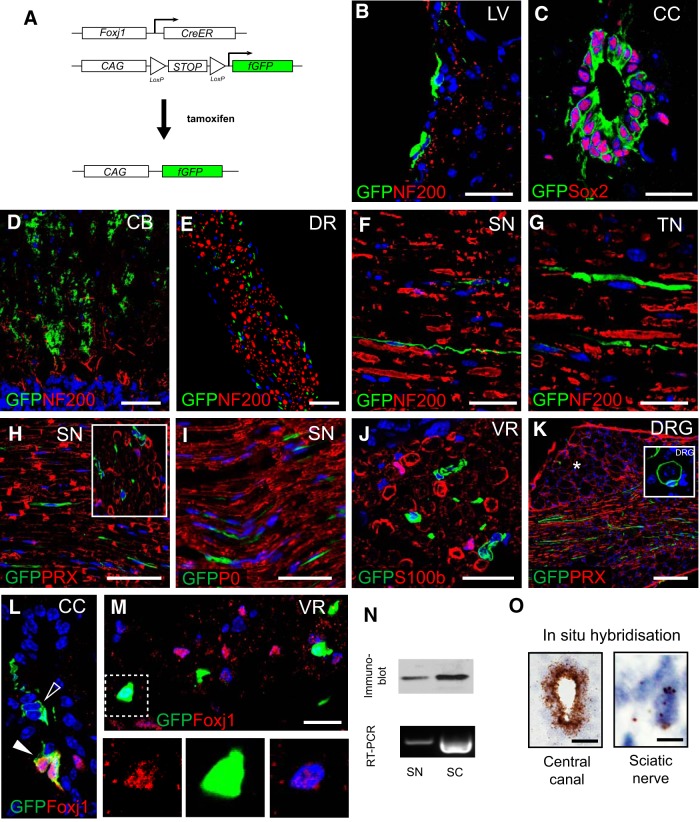
Foxj1 controlled GFP expression labels cells in both the CNS and PNS. ***A***, Diagram illustrating transgene design of Foxj1 promoter-controlled, tamoxifen-induced activation of fGFP reporter gene expression. ***B***–***K*** are images from multiple immunostaining for GFP and different cell markers. GFP-expressing cells are detected in ependymal cells lining lateral ventricles (LV; ***B***); ependymal cells in the central canal of spinal cord (CC; ***C***), which coexpress Sox2; and Bergmann glia in cerebellum cortex (CB; ***D***). Foxj1-controlled GFP is also expressed in the PNS. GFP^+^ cells are scattered between neurofilament-positive axons in spinal dorsal roots (DR) (***E***, transverse section), sciatic nerves (SN, ***F***, longitudinal section), and trigeminal nerves (TN) (***G***, longitudinal sections). GFP-expressing cells do not colocalize with myelinating SC markers (***H***, inset shows transverse view), P0 (***I***), and S100β (***J***, transverse section). Image in ***K*** is from a dorsal root ganglion (DRG) showing GFP-expressing cells among nerve fibers but few among neuronal cell bodies (asterisk). Occasionally, Foxj1-GFP cells surround a DRG neuron at axonal entry zone (inset in ***K***). Image ***L*** illustrates immunoreactive Foxj1^+^ cells in small number of ependymal cells in CC, which also expressed GFP (solid arrowhead). However, not all GFP^+^ are detected with Foxj1^+^ (open arrowhead). Nucleus-localized Foxj1 is detectable in the transverse section of ventral root (VR) of spinal cord in GFP^+^ or GFP^−^ cells (***M***). The blue color in all images is the DAPI-stained nucleus. Foxj1 protein and mRNA can be detected in peripheral nerves as well as spinal cords (SC) by Western blot and RT-PCR (***N***). Foxj1 transcript can be visualized by the sensitive RNAScope technique in sciatic nerve and spinal cord central canal (***O***). Scale bars in the images indicate 20 μm except for ***E***, which is 50 μm, and ***K***, which is 100 μm.

Mice with C57BL6 background expressing Foxj1-CreERT2 (Rawlins et al., 2007), PDGFRa-CreERT2 (Rivers et al., 2008), and Sox10-CreERT2 (Laranjeira et al., 2011; Zhao et al., 2015) were crossed with Cre-dependent reporter mouse lines expressing farnesylated GFP (Rawlins et al., 2009) (Rosa-CAG-fGFP) to produce double heterozygous mice, denoted as *Foxj1-GFP*, *PDGFRa-GFP*, and *Sox10-GFP*, respectively (Fig. 1*A*). Mice of either sex have been used in this study. For each experimental group, three to nine animals were used; the exact numbers used in quantification are provided in the Results section. All animal studies were conducted under the Animals (Scientific Procedures) Act 1986 Amendment Regulations 2012 following ethical review by the University of Cambridge Animal Welfare and Ethical Review Body.

##### Tamoxifen administration and surgery.

To induce Cre-mediated recombination, mice at the age of 7–8 weeks were administered with tamoxifen prepared in corn oil (Sigma-Aldrich) at 250 mg/kg daily for 4 consecutive days by oral gavage. Subsequent procedures were performed 8–10 d following the final tamoxifen administration. All surgical procedures were conducted under aseptic conditions and the animals were provided perioperative analgesia (buprenorphine 0.05 mg/kg, i.p.) and additional care. CNS demyelination lesions were created in dorsal or ventral spinal cord white matter by direct injection of lysolecithin as described previously (Zhao et al., 2008). Briefly, under isoflurane anesthesia, the dorsal surface of thoracolumbar spinal cord was exposed by removing the soft tissue between vertebra between T13 and L1. One microliter of lysolecithin (1% in PBS) was injected into dorsal or ventral funiculus via a 10 μl Hamilton syringe with a fine glass tip from pulled capillary attached.

Sciatic nerve crush and transection injuries were induced under isoflurane anesthesia. A 0.5 cm length of mouse sciatic nerve was exposed at the level of midfemur. A crush injury was created by clamping the entire nerve with a pair of fine forceps (Dumont #5 at a marked position ∼3 mm from the tip) for 20 s twice. Sciatic nerve transection was conducted by cutting the nerve with a pair of fine scissors (10.5 cm, straight). The intact contralateral nerve served as a control.

Mice were killed by injection of 20% pentobarbital (Pentoject) followed by transcardiac perfusion fixation with 4% paraformaldehyde prepared in PBS, pH 7.4. The required tissue was removed and further immersion-fixed with the same fixative for 2 h at room temperature, followed by treatment with 20% sucrose prepared in PBS at 4°C overnight. Frozen sections were cut at 12 μm for image analysis following immunohistochemistry or *in situ* hybridization.

##### Immunohistochemistry.

Frozen sections of 12 μm thickness were subject to a standard protocol for immunofluorescence staining as described previously (Zhao et al., 2008). Where required, heat-mediated antigen retrieval was performed using a commercial antigen retrieval solution (Sigma-Aldrich). The following antibodies were used: goat /rabbit anti-GFP (Abcam), rabbit anti-Olig2 (Millipore), rabbit anti-GFAP (Dako), rabbit anti-periaxin (gift from Professor Peter Brophy or from Sigma-Aldrich), rabbit anti-S100β (Dako), rat anti-PDGFRa (CD140a; BD Bioscience), rabbit anti-prolyl-4 hydroxylase β (P4HB; Abcam), rabbit anti-HSP47 (BioVision), rabbit anti-IBA1 (Wako), rabbit anti-smooth muscle actin (SMA; Abcam), rabbit anti-Ki67 (Abcam), chicken anti-myelin protein zero (P0) (Abcam), goat anti-Sox2 and goat anti-Sox10 (Santa Cruz Biotechnology), rat anti-CD31 (BD Biosciences), rabbit anti-fibronectin (Millipore), rat anti-L1cam (Millipore), and rabbit anti-Foxj1 (Insight Biotechnology) Secondary antibodies against relevant primary antibodies labeled with either Alexa Fluor 488 or Alexa Fluor 594 were from Thermo Fisher Scientific. The images were acquired with a Leica SP5 confocal microscope or a Zeiss Axio Observer A1 fluorescence Imaging System.

##### *In situ* hybridization.

Expression of Foxj1 was examined using single-plex RNAscope *in situ* hybridization (chromogenic). The mouse Foxj1 probe and all reagents were obtained from ACDBio (https://acdbio.com/) and the hybridization and visualization were performed on frozen sections from paraformaldehyde-fixed animals according to the manufacturer's protocol.

##### RT-PCR.

Fresh pieces of spinal cord or sciatic nerve were dissected out from normal wild-type mice 8–9 weeks old following euthanasia. Total RNA were extracted using RNeasy mini kit and cDNA was prepared using the QuantiTech Reverse Transcription kit (all from Qiagen), which incorporated a genomic DNA wipe-out step. Conventional PCR was performed using a commercial PCR mix (MegaMix Blue; Cambio). PCR products from spinal cord and sciatic nerve were verified by sequencing.

##### Immunoblot.

Spinal cord and sciatic nerves were harvested as for RT-PCR. Protein extraction was performed using CelLytic MT Cell Lysis buffer (Sigma-Aldrich) supplemented with protease inhibitor mixture. Equal amounts of protein were denatured in sample buffer and resolved on 4–12% SDS-polyacrylamide gels (Invitrogen). Foxj1 was detected using mouse anti-foxj1 (Thermo Fisher Scientific) and visualized with ECL Plus (GE Healthcare).

##### Pre-embedding immunogold labeling electron microscopy.

Animals administered with tamoxifen for fate mapping were fixed by perfusion via the left ventricle with 3% PFA and 0.5% glutaraldehyde in PBS. After washing with PBS, segments of sciatic nerve and spinal cord were embedded with 4% low-melting-point agarose and sliced at 100 μm on a vibratome (Leica). Pre-embedding immunogold labeling was performed according to the manufacturer's protocol (Aurion). Briefly, following permeabilization and blocking, the tissue slices were incubated at 4°C with goat anti-GFP antibody (Abcam) for 48 h, followed by ultrasmall gold particle-conjugated anti-goat IgG (Aurion) for 48 h at 4°C. The samples were then subjected to a standard resin-embedding protocol incorporating a silver enhancement step after osmium tetroxide (0.5%) treatment. The ultrathin sections were examined on a Hitachi H600 transmission electron microscope.

##### Quantification.

Immunolabeled cells were quantified by counting the positive cells in which the nucleus was also stained with DAPI on sections. Data are presented as mean ± SE as the relative proportion of two labeled populations. A minimum of three animals have been included in each experimental group. To compare cell proportion in dorsal and ventral spinal roots, transverse sections were used for cell counts, and Student's *t* test was used for statistical analysis.

## Results

### Foxj1 labels CNS ependymal cells and nonmyelinating cells in peripheral nerve

Consistent with previous studies (Yu et al., 2008; Muthusamy et al., 2014), tamoxifen administration in mice expressing Foxj1 fate-mapping reporter construct (Foxj1-GFP; Fig. 1*A*) led to induction of GFP expression in ependymal cells lining the cerebral ventricles and central canal in the spinal cord (Fig. 1*B*,*C*). Labeled cells were also found in other parts of brain including the molecular layer of cerebellum (Fig. 1*D*). In the spinal cord labeling was restricted to the ependymal cells of the central canal. Labeled cells were also detected in dorsal and ventral spinal roots (Fig. 1*E*) and throughout the sciatic and trigeminal nerves (Fig. 1*F*,*G*). We found on an average of 46.41 ± 8.79% (*n* = 9) of ependymal cells were labeled with GFP in tamoxifen inducible Foxj1-GFP mice. Since Foxj1 expression appears to be highest in ependymal cells in the nervous system, we expect the induction efficiency would be similar or lower in other cell types in CNS and peripheral nerves.

The findings prompted us to characterize the distribution and identity of the Foxj1 labeled (Foxj1^+^) cells in the PNS by immunohistochemistry using specific markers for each major cell type. As illustrated in Figure 1, *F–I*, GFP^+^ cells in longitudinal sections of sciatic nerve had an elongated morphology with oval-shaped nuclei. These cells did not surround axons as revealed by double immunostaining for neurofilament, as an axonal marker (Fig. 1*E–G*), and did not have overlapping expression with myelinating SC-specific markers, periaxin (PRX), myelin protein zero (MPZ), and S100β (Fig. 1*H–J*), indicating that the reporter protein GFP was not expressed by myelinating SCs. Foxj1 labeled cells were occasionally found in the dorsal root ganglia (DRG), but far less abundantly than in peripheral nerves (Fig. 1*K*). We found that occasionally Foxj1-GFP^+^ cells were present at the edge of DRG, with processes wrapping DRG neurons (Fig. 1*K*, inset), but these were rare. To determine whether the Foxj1 promoter-driven GFP expression reflected the expression of Foxj1 itself, we examined the level of Foxj1 protein by immunohistochemistry, Western blot, and mRNA with RT-PCR in peripheral nerves. Immunostaining only detects a small number of ependymal cells in the central canal, not in all GFP-expressing cells (Fig. 1*L*). In the ventral root of the spinal cord, nucleus-localized Foxj1^+^ cells were detected and the intensity of staining was weak (Fig. 1*M*). The lower tissue level of expression in sciatic nerve was consistent with Western blot and RT-PCR compared with spinal cord tissue (Fig. 1*N*). A very low level of Foxj1 mRNA expression was further confirmed with *in situ* hybridization using the sensitive RNAScope technique (Fig. 1*O*).

### Foxj1-expressing cells in peripheral nerve are a subpopulation of nonmyelinating SCs with fibroblast-like features

**Figure 2.**
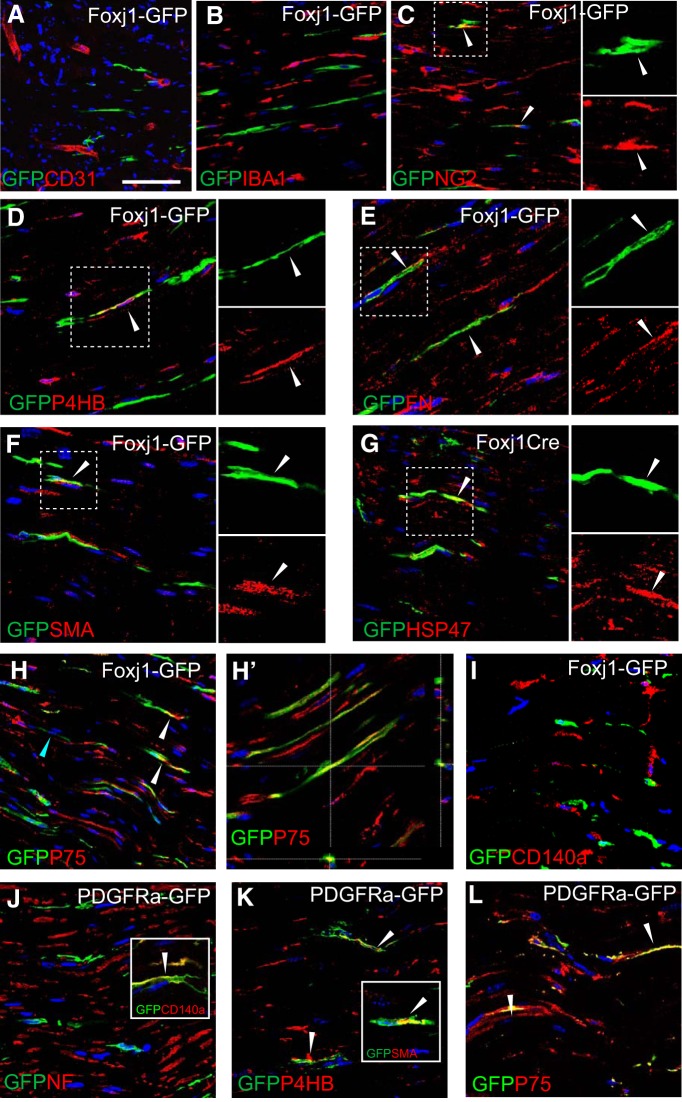
Foxj1-GFP-labeled cells express fibroblast markers but are of a distinct population from PDGFRa-GFP labeled cells in peripheral nerves. Immunofluorescence characterization of normal sciatic nerves (SN) from Foxj1-GFP mice treated with tamoxifen. Double immunostaining for GFP and various markers was performed on longitudinal sections. Colocalized staining is marked with arrowheads. Overlaid images show that Foxj1 labeled cells in SN are not associated with endothelial cells labeled by CD31 (***A***), nor do they express the macrophage marker IBA1 (***B***). A proportion of GFP^+^ cells are labeled with NG2 (***C***) and proteins usually expressed by fibroblasts such as fibronectin (FN, ***D***), P4HB (***E***), SMA (***F***), and HSP47 (***G***). In ***D***–***G***, the split channel views of the boxed area by dotted lines are shown in separate images. Foxj1-GFP-labeled cells also expressed P75NTRs (***H*** and confocal orthogonal view in ***H′***). GFP-labeled cells do not coexpress PDGFRa (CD140a, ***I***). PDGFRa fate-mapping GFP reporter mice were characterized by double immunostaining. PDGFRa-GFP labeled cells are not associated with neurofilament-labeled axons (***J***). The GFP^+^ cells are confirmed expressing CD140a (inset in ***J***) and are double labeled with P4HB and SMA (***K*** and inset in ***K***). PDGFRa-GFP-labeled cells are also detected for P75NTR (***L***). Scale bar indicates 50 μm for all images.

We next performed a series of double-labeling immunohistochemistry experiments to determine the identity of Foxj1-labeled cells in peripheral nerves. In addition to axons and myelinating SCs, the peripheral nerves contain endothelial cells, pericytes, residential macrophages, fibroblast-like cells, and nonmyelinating SCs, which group small diameter axons into Remak bundles. Double-labeling immunofluorescence staining showed that GFP was not expressed by CD31-labeled endothelial cells (Fig. 2*A*), nor by macrophages marked by ionized calcium-binding adapter molecule 1 (IBA1; Fig. 2*B*). A proportion of Foxj1-GFP-labeled cells (<10% GFP^+^ cells) were found to express NG2 (Fig. 2*C*), which has been associated with endoneurial fibroblast-like cells in the peripheral nerves (Richard et al., 2012, 2014). Further staining revealed that GFP expression colocalized with a number of other fibroblast-expressed molecules such as fibronectin, prolyl-4-hydroxylase B (P4HB), smooth muscle actin (SMA), and heat shock protein 47 (HSP47) (Fig. 2*D–G*). However, a considerable proportion (73.4% ± 12.7%) of GFP^+^ cells also express the p75 neurotrophin receptor (P75NTR) (Fig 2*H*,*H′*), which is regarded as a marker specific for SC precursors and nonmyelinating SCs (NMSCs). Foxj1-GFP^+^ cells did not express CD140a (Fig. 2*I*), also known as platelet-derived growth factor receptor α (PDGFRa), which we and others have previously shown to mark fibroblasts in peripheral nerves (Raivich and Kreutzberg, 1987; Zawadzka et al., 2010). We compared the distribution of labeled cells in sciatic nerve of PDGFRa-driven fate-mapping mice and found that most PDGFRa-GFP cells also expressed CD140a, as expected (Fig. 2*J*, inset), as well as P4HB and SMA (Fig. 2*K*, inset). Unexpectedly, PDGFRa-GFP cells were found to be labeled by P75 (Fig. 2*L*). Therefore, even though Foxj1- and PDGFRa-labeled cells both can express fibroblast markers, they are mutually exclusive with regard to the expression of CD140a and therefore likely belong to separate populations in adult peripheral nerves.

**Figure 3.**
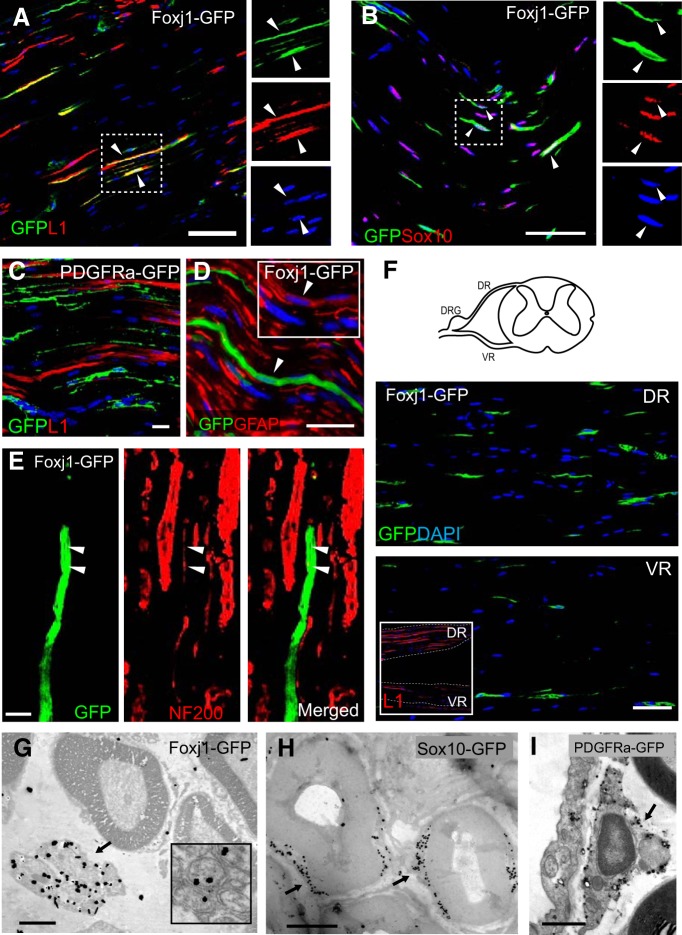
Foxj1 labels nonmyelinating SCs. Images in ***A***–***F*** illustrate merged double immunofluorescence staining on longitudinal sections of sciatic nerve (SN) from adult Foxj1-GFP mice treated with tamoxifen. Most GFP-expressing cells coexpress the L1 cell adhesion molecule, a marker of nonmyelinating SCs in peripheral nerves (***A***). GFP-expressing cells can also express Sox10, a neural crest-related transcription factor (***B***). The single channel images of the same area in ***A*** and ***B*** marked by dotted line are shown in image sets on the right side of the main images. PDGFRa-labeled cells (PDGFRa-GFP) in sciatic nerves do not coexpress L1 (***C***). Low levels of GFAP are detected in Foxj1-GFP-expressing cells (***D***, inset). Costaining for GFP and neurofilament reveals a close association between GFP and small-diameter axons (arrowheads) (***E***). Dorsal and ventral roots (DR and VR, respectively) from lumbar spinal cord were stained for GFP, showing greater numbers of Foxj1-GFP^+^ cells in dorsal root (***F***) and this was correlated with the proportion of L1 immunoreactivity in corresponding locations (inset in ***F***). ***G***–***I*** are images from pre-embedding immunogold electron microscopy against GFP on SN from Foxj1 and Sox10 fate-mapping mice. Silver-enhanced gold particles were mainly detected on “Remak” bundles in Foxj1-GFP samples (***G***), whereas the majority of gold particles in Sox10 fate-mapping mice are deposited in SC cytoplasm around the myelinated axons but excluded from compact myelin sheaths (***H***). PDGFRa labeling identifies endoneurial fibroblast like cells (***I***). Scale bars: ***A***–***E***, 25 μm; ***F***, 50 μm; ***G***, 2 μm; ***H***, ***I***, 1 μm.

We next performed further characterization to verify whether Foxj1 was expressed by NMSCs (Remak cells) using additional markers. Most Foxj1-GFP-labeled cells (77.8% ± 5%) in normal adult sciatic nerve colocalized with L1 cell adhesion molecule (Fig. 3*A*), a protein expressed by immature myelinating cells and NMSCs in peripheral nerves and some neurons (Nieke and Schachner, 1985; Samatov et al., 2016). No PDGFRa-GFP-labeled cells were detected to coexpress L1 (Fig. 3*C*). Foxj1-GFP cells also expressed Sox10 (Fig. 3*B*), a neural crest transcription factor and pan-SC lineage marker (Nonaka et al., 2008), as well as glial fibrillary acidic protein (GFAP), an intermediate filament found in NMSCs (Fig. 3*D*) (Jessen et al., 1990). These observations strongly suggested that Foxj1 expression labels a subpopulation of NMSCs, a conclusion supported by the observations that GFP^+^ cells were found in close association with small caliber, neurofilament-positive axons in a manner that resembled Remak bundles (Fig. 3*E*) and considerably more Foxj1-GFP^+^ cells were detected in dorsal roots compared with ventral roots of spinal cord, the former containing a higher proportion of nonmyelinated fibers (Griffin and Thompson, 2008; He et al., 2012) (Fig. 3*F*). However, there was no significant difference in the percentage of L1-expressing GFP^+^ cells between dorsal and ventral spinal root (87.38 ± 3.95%, *n* = 5 vs 81.51 ± 5.86, *p* = 0.447). Quantification showed that less than half (40.55 ± 10.46%, *n* = 6) of L1^+^ cells in SN were labeled for GFP, though this proportion is likely under estimated considering the level of inducible recombination efficiency. We then confirmed the expression of Foxj1 likely in NMSCs by immunogold electron microscopy: the silver enhanced gold particles conjugated with GFP antibody were found clearly concentrated in Remak bundles (Fig. 3*G*), whereas in Sox10-GFP mice, labeling was mainly in the cytoplasm of myelinating SCs (Fig. 3*H*). In PDGFRa-GFP mice, the gold particles were found in NMSCs that resembled fibroblast-like cells (Fig. 3*I*).

**Figure 4.**
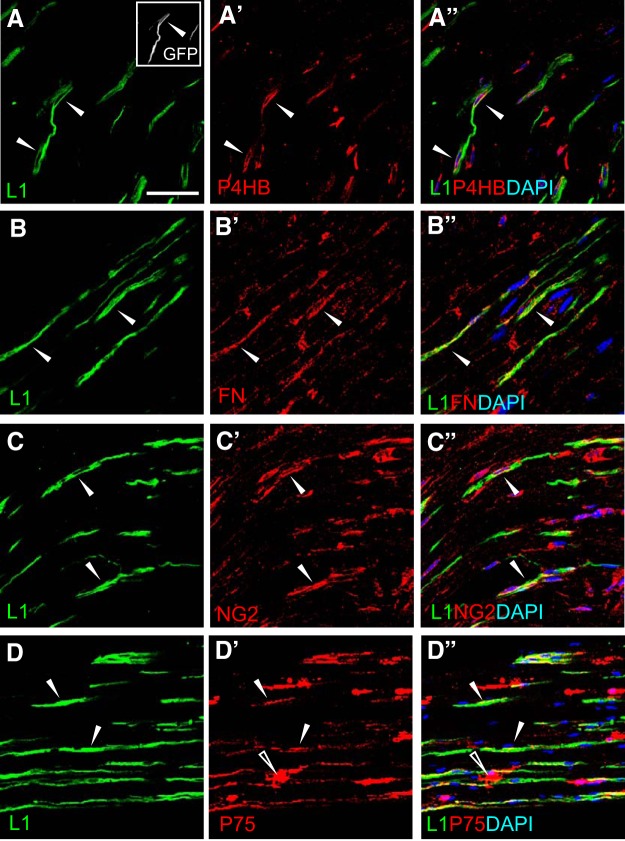
Nonmyelinating SCs express fibroblast markers. Image sets ***A***–***C*** illustrate immunostaining of longitudinal sections from unlesioned sciatic nerves for L1Cam (L1, green), a marker labeling nonmyelinating SCs, with markers labeling peripheral nerve fibroblasts (red). The merged images show that a proportion of L1 immunoreactive cells colocalize with P4HB (***A***–***A″***), fibronectin (FN, ***B***–***B″***), and NG2 (***C***–***C″***). Image set ***D*** shows that the majority of L1-labeled cells are also positive for P75NTR (P75) (***D′*** and merged image ***D″***) and a proportion of P75^+^ cells are not labeled with L1. The open arrowheads highlight a P75^+^ cell that is not labeled with L1 (***D′*** and ***D″***). The solid arrowheads in all images indicate examples of colocalization. Scale bar indicates 50 μm, applicable to all images.

The results indicate that Foxj1-GFP^+^ cells express markers for fibroblasts and NMSCs, raising the possibility that either Foxj1 labels both populations or these marker lack specificity in peripheral nerves. To resolve this, we further characterized unlesioned peripheral nerves by immunostaining with several selected markers for fibroblasts and the NMSC marker L1. We use L1 as an NMSC marker because almost all L1^+^ cells also express p75 (97.39 ± 0.7%, *n* = 6) and a similar proportion of P75^+^ cells expressed L1 (73.69 ± 4.63%, *n* = 6). Many of the L1^−^ fraction of P75^+^ cells exhibited a different morphology from the typical elongated shape of L1^+^ cells (Fig. 4*D*), suggesting that these cells may account for the fibroblast-like population labeled by PDGFRa (Fig. 2*L*). As shown in Fig. 4, L1 was coexpressed with P4HB, fibronectin, and NG2. Quantification indicated that the overlapping cells only accounted for ∼25% of L1^+^ cells, which were colabeled with either P4HB (25.37 ± 6.46%, *n* = 6) or NG2 (16.5 ± 5.23%, *n* = 3). Similarly, <20% of either P4HB^+^ cells (17.21 ± 2.57%, *n* = 6) or NG2^+^ cells (17.52 ± 3.08%, *n* = 3) also expressed L1. Triple-labeled cells that were also L1^+^ and GFP^+^ were detected with all of the fibroblast markers tested. The results confirmed that a population of NMSCs express markers used to label fibroblasts in various tissues.

### Peripheral nerve-derived Foxj1^+^ cells but not ependymal cells contribute to CNS SC remyelination

**Figure 5.**
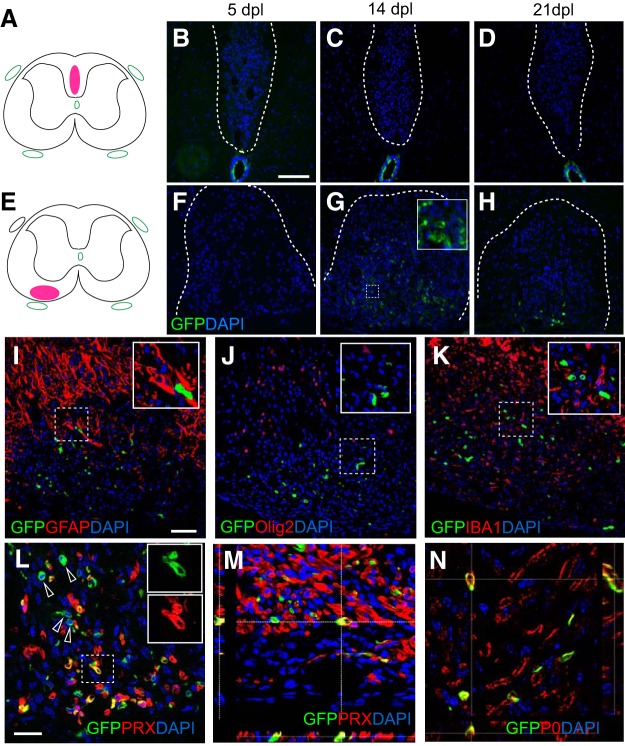
Foxj1-labeled cells give rise to remyelinating SCs in demyelinating lesions in spinal cord white matter. Demyelination lesions were induced in mice Foxj1-GFP-expressing mice by direct injection of lysolecithin in either dorsal or ventral funiculi as shown in ***A*** and ***E***, with corresponding images from GFP immunostaining for 5, 14, and 21 dpl. Few GFP-expressing cells have been detected in dorsal lesions (***B***–***D***), whereas a large number of GFP^+^ cells are found in ventral lesions from 14 dpl (***F***–***H***). Double immunostaining indicates that Foxj1-GFP cells do not express the astrocyte marker GFAP (***I***), the oligodendrocyte lineage marker Olig2 (***J***), nor the microglial marker IBA1 (***K***). ***L*** shows that considerable numbers of GFP-expressing cells coexpress the myelinating SC marker PRX (arrows). The box area is shown as split channels (***L1*** and ***L2***) and merged in (***L3***). ***M***, Orthogonal view of confocal images of double immunostaining confirming the colocalization of GFP and PRX in ventral lesions at 14 dpl. The SC identity of GFP^+^ cells in lesion is verified by colocalization with myelin P0, as illustrated by orthogonal confocal view in ***N***. Insets in the images show magnified area in lesions in respective images marked with dotted outlines, for either double staining (***I***–***K***) or single colors (***G***, ***L***). Scale bars in ***B*** indicate 100 μm and apply to ***C***, ***D***, and ***F***–***H***. Scale bar in ***I*** indicates 50 μm and applies to ***J*** and ***K***. In ***L***–***N***, the scale bars indicate 25 μm.

Next, we investigated whether Foxj1-GFP-labeled cells contribute to remyelination in the CNS. We performed spinal cord focal demyelination by injecting lysolecithin into the white matter of tamoxifen-treated mice. Discrete, focal lesions were created in either the dorsal or ventral white matter funiculi of the thoracolumbar spinal cord (Fig. 5*A–H*). Within dorsal lesions, very few GFP-expressing cells were detected at 5, 14, and 21 d postlesions (dpl) despite their close vicinity to the central canal in which ependymal cells were labeled (Fig. 5*A–D*). However, considerable numbers of GFP-expressing cells were present in ventral lesions at both 14 and 21 dpl, when remyelination is in progress or near completion. The labeled cells were mainly located in the center of the lesion (Fig. 5*G*,*H*). To identify the fate of GFP^+^ cells following demyelination, we performed immunohistochemistry for specific markers of CNS glial cells: Olig2 for oligodendrocyte lineage cells, GFAP for astrocytes, and IBA1 for microglia/macrophages. No GFP^+^ cells expressed any of these markers, indicating that Foxj1^+^ cells do not give rise to CNS cell types in the white matter (Fig. 5*I–K*). The distribution of GFP^+^ cells, however, resembled that previously described for remyelinating SCs (Zawadzka et al., 2010). The majority of the GFP-expressing cells present in the lesion at 14 dpl also expressed PRX (Fig. 5*L*,*M*), a marker only expressed in myelinating SCs (Gillespie et al., 1994; Zawadzka et al., 2010). It is also confirmed by double immunostaining with P0 (Fig. 5*N*). Therefore, Foxj1^+^ cells can give rise to remyelinating SCs following CNS demyelination. There was considerable variation in the numbers of Foxj1-derived SCs: among the nine mice with ventral lesions, five contained GFP^+^ cells in the demyelinated area. The proportion of GFP-labeled PRX^+^ cells over all PRX^+^ SCs ranged from none to ∼33%. An average of 54.18 ± 12.08% (*n* = 5) of GFP^+^ cells in the ventral lesions at 14 dpl coexpressed PRX and most of the PRX^−^GFP^+^ cells resembled the morphology of PRX^+^ cells (Fig. 5*L*). In dorsal and some ventral spinal cord lesions, despite of the absence of Foxj1-labeled cells following demyelination, remyelinating SCs were invariably detected, which were most likely generated from OPCs.

**Figure 6.**
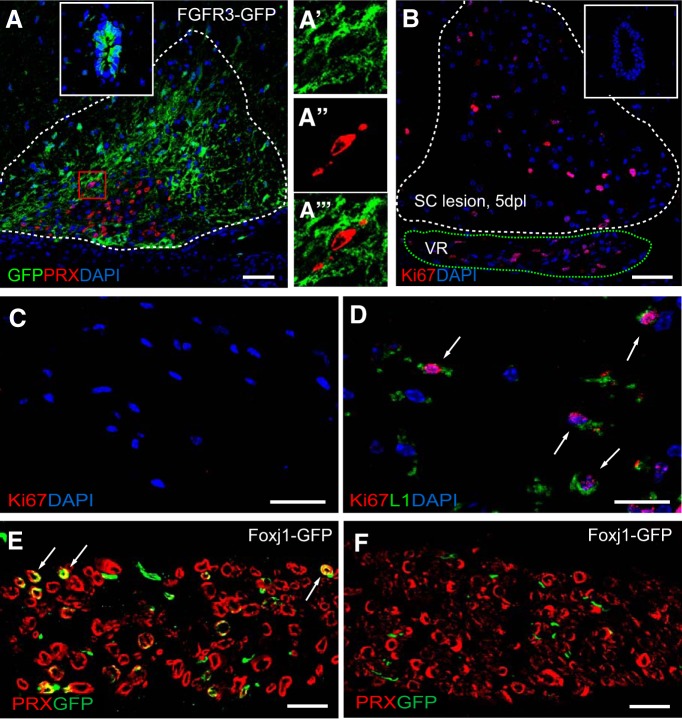
SC remyelination from Foxj1-GFP-labeled cells in spinal cord lesions are not derived from ependymal cells, but likely originate from peripheral nerves. ***A***, Demyelinated lesion at 21 dpl in ventral spinal cord white matter of a FGFR3-GFP reporter mouse treated with tamoxifen. Image shows overlaid double immunostaining for GFP and PRX, with the dotted line marking the boundaries of the lesion. GFP is expressed by ependymal cells lining the central canal of the spinal cord (inset). A magnified area with in the red box in the lesion in ***A*** showing separate and overlaid channels indicating nonoverlapping expression of GFP and PRX is shown in ***A′***, ***A″***, and ***A‴***). ***B*** illustrates a ventral spinal cord lesion (white dotted line) at 5 dpl immunostained for the proliferation marker Ki67. Ki67^+^ cells are found in the adjacent ventral roots (VR, green dotted line), but not in ependymal cells in central canal (inset). There are no Ki67^+^ cells in the contralateral VR (***C***). The Ki67^+^ nucleus are found in L1^+^ cells in the VR of ventral spinal cord lesion side (***D***). ***E*** and ***F*** illustrate Foxj1-GFP cells colabeled with PRX in the VR of spinal cord lesion side at 14 dpl, but none in the VR of contralateral side. Scale bars indicate 100 μm in ***A***, 50 μm in ***B***, and 20 μm in ***C***–***F***.

To establish the origin of Foxj1-GFP^+^ CNS remyelinating SCs, we first investigated whether they might be derived from Foxj1^+^ ependymal cells. We used a FGFR3-GFP mouse reporter line, in which GFP is expressed by both ependymal cells and astrocytes (Zawadzka et al., 2010). In ventral lesions, there was an abundance of GFP^+^GFAP^+^ astrocytes in and around the lesion and surrounding a central region containing many PRX-expressing SCs: however, none of these SCs coexpressed GFP (Fig. 6*A*). Many of the GFP^+^ cells within the ventral lesion were located around the CNS–PNS transitional region (Fig. 6*L*,*M*). This led us to conclude that the GFP^+^ CNS remyelinating SCs in Foxj1-GFP fate-mapping mice were derived from Foxj1^+^ cells present within peripheral nerves. This conclusion was supported by the occurrence of Ki67^+^-proliferating L1^+^ cells in ventral roots adjacent to ventral white matter lesions at 5 dpl (but not on the contralateral unlesioned side) (Fig. 6*B–D*), indicating a CNS injury induced response. There was no Ki67^+^ ependymal cells found in central canal in the spinal cords with white matter lesions (Fig. 6*B*, inset). In addition, all ventral lesions containing Foxj1-GFP cells (five of nine animals) were directly adjacent to ventral spinal roots in which a direct injury from the lesion induction likely occurred because a small number of GFP-labeled PRX^+^ were also found, but were not present in the contralateral side (Fig. 6*E*,*F*).

### Foxj1-expressing cells in peripheral nerves respond to peripheral nerve injury and give rise to repair SCs

**Figure 7.**
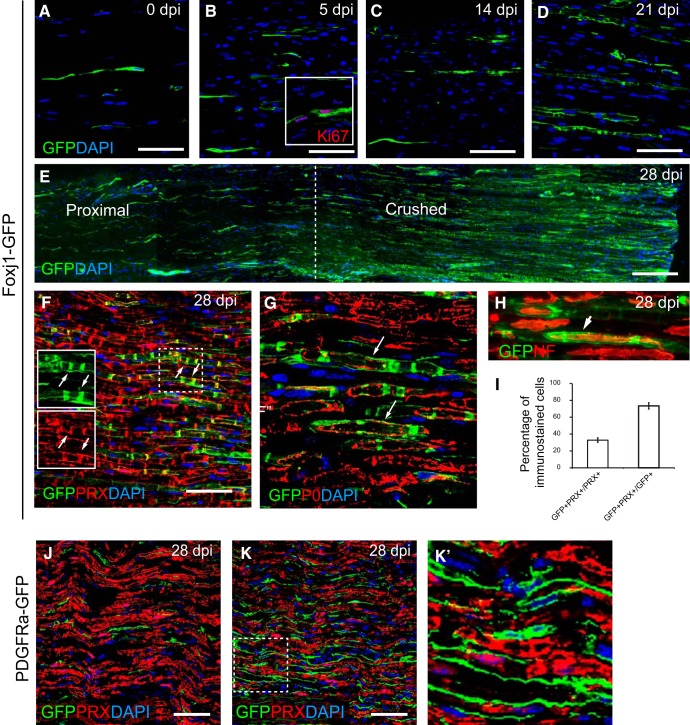
Foxj1-GFP-labeled cells become repair SCs following sciatic nerve injury. Sciatic nerve crush lesions were created in adult mice expressing reporter genes and samples were analyzed by immunofluorescence staining. ***A***–***D*** show an area of sciatic nerve in longitudinal section immunostained to reveal Foxj1-GFP-labeled cells in normal (***A***) and crushed nerve at 5, 14, and 21 dpi (***B***–***D***, respectively). Inset in ***B*** illustrates GFP^+^ cells expressing Ki67. ***E*** is a montage combined from a series of overlapping images spanning the proximal and crushed site at 28 dpi. Doubling immunostaining shows that, at 28 dpl, GFP colocalizes with the mature myelinating SC marker PRX (***F***). The dotted outlined area is shown in single channels as in insets in ***F***, with examples of colocalization marked with arrows. GFP labels the myelinated axons marked by myelin P0, which appears only localized in the cytoplasm rather than compact myelin (***G***). GFP-labeled cells enclose the axons (arrows) marked by neurofilament (NF) staining (***H***). Approximately 30% of remyelinated axons in the crush area are labeled with GFP, but >70% of GFP immunoreactivity colocalizes with PRX (mean ± SE, *n* = 3; ***I***). ***J*** and ***K*** show GFP and PRX double immunostaining in control (uninjured nerve) and crushed sciatic nerve from PDGFRa-GFP animals at 28 dpi. The magnified boxed area in ***K*** is shown in ***K′*** which shows that there is no colocalization between GFP and PRX. Scale bars indicate 50 μm for all images.

The ability of Foxj1^+^ cells in peripheral nerve to contribute to CNS remyelination prompted us to examine their response to peripheral nerve injury. Following sciatic nerve crush, there was an increase in GFP^+^ cells within lesioned area (Fig. 7*A–E*) associated with the expression of the proliferation marker Ki67 (Fig. 7*B*, inset). At 21 and 28 d postinjury (dpi), many GFP cells displayed a morphology resembling myelinating SCs with distinct appearance of cytoplasmic bands (Fig. 7*D*,*E*). Double labeling with the myelinating SC marker PRX confirmed a close colocalization (Fig. 7*F*). As in CNS lesions, GFP also surrounded myelin labeled with antibodies to P0 (Fig. 7*G*). Further staining with neurofilament antibody confirmed GFP ensheathing axons in a manner suggestive of myelin sheath formation (Fig. 7*H*). Quantification of the lesion area at 28 dpi indicated that ∼30% of PRX^+^ cells were also GFP^+^ and nearly 80% of GFP-labeled cells coexpressed PRX (Fig. 7*I*), suggesting that most Foxj1^+^ cells associated with the lesion became myelinating SCs. As a control, we performed crush injury on PDGFRa-GFP mice. There was a considerable increase in GFP labeled cells at 28 dpi compared with controls (Fig. 7*J*,*K*). However, there was no overlap between GFP and PRX immunoreactivity (Fig. 7*K*), confirming that PDGFRa-GFP cells did not give rise to new SCs. This observation is consistent with previous reports that PDGFRa-labeled cells in the PNS do not make SCs (Zawadzka et al., 2010; Assinck et al., 2017).

**Figure 8.**
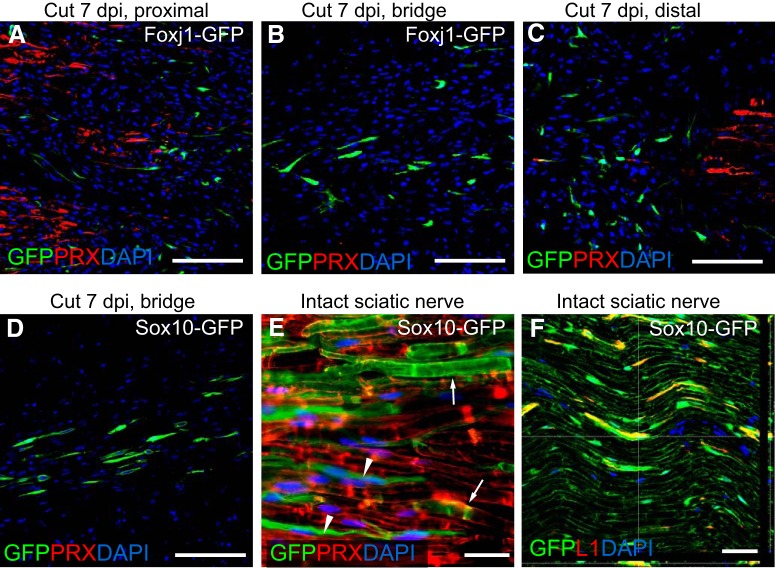
Early-appearing SC lineage cells following nerve transection are mainly NMSCs. Immunohistochemistry for GFP and PRX was performed on longitudinal sections of sciatic nerves from mice that received transection injury. Shown is an area of proximal stump (***A***), rejoining bridge (***B***), and distal stamp (***C***) following sciatic cut 7 dpi from a tamoxifen-treated Foxj1-GFP mouse. Staining of GFP-expressing cells at the bridge at the same time point following transection from a Sox10-GFP mouse is shown in ***D***. ***E*** shows an area of intact sciatic nerve from Sox10-GFP mice indicating that both myelinating (arrows) and nonmyelinating cells (arrowheads) have been labeled by GFP. The GFP-labeled cells in Sox10-GFP sciatic nerve coexpress L1, as shown in confocal image in ***F***, confirming their identity as nonmyelinating SCs. Scale bars indicate 100 μm in ***A***–***D*** and 50 μm in ***E*** and ***F***.

We further examined the response of Foxj1-labeled cells in a sciatic nerve transection model. Following transection, a connective tissue bridge forms between the proximal and distal stumps that contains macrophages, fibroblasts, endothelial cells, and NMSCs (McDonald et al., 2006). At 7 dpi, the numbers of Foxj1^+^ cells were increased in the proximal and distal stumps (Fig. 8*A*,*C*). The cells had an elongated bipolar spindle shape and were aligned in the direction of the nerve longitudinal axis. Foxj1^+^ cells were present in the bridge (Fig. 8*B*). The numbers of GFP^+^ cells in the bridge were similar to those identified in samples from Sox10-GFP reporter mice that were also subjected to nerve transection (Fig. 8*D*). Because Sox10-GFP labels both myelinating and nonmyelinating SCs before injury induction (Fig. 8*E*,*F*), one would predict significantly more GFP^+^ cells in injury area. That there were not suggested that NMSCs may be the predominant SCs responding to injury at early stage following injury. To provide direct evidence for the relative contribution of myelinating and nonmyelinating SCs to the regeneration of PNS injury, definitive cell-specific fate mapping using transgenic fate-mapping mouse lines of equal recombination efficiency would be necessary.

## Discussion

Remyelination in the CNS is usually achieved by newly generated oligodendrocytes derived from CNS progenitor cells (often called OPCs). In some circumstances, especially when there is concurrent loss of astrocytes, remyelinating SCs also occur. Fate-mapping data suggest that the predominant source of the both new oligodendrocyte and SCs are PDGFRa/NG2-expressing OPCs, which are actively recruited into the demyelinated area. Neither astrocytes nor mature oligodendrocytes are sources of new remyelinating cells (Zawadzka et al., 2010; Crawford et al., 2016). However, other cell types may also contribute to remyelinating cell population, such as ependymal cells (EPCs) lining the ventricles and central canal of spinal cord. In this study, we addressed the question by the genetic fate-mapping to label Foxj1-expressing EPCs and follow their distribution at different stages of remyelination following lysolecithin-induced demyelination in the spinal cord. Foxj1, a master regulator in the formation of motile cilia, is a specific marker of ciliated cells such as lung epithelial cells and EPCs. In this study, we found that Foxj1 is also expressed by a population of NMSCs and that these cells can give rise to remyelinating SCs in the CNS.

Our discovery of remyelinating CNS SCs derived from Foxj1-expressing cells raised the question from exactly which Foxj1 population these cells originated. Several studies have reported that EPCs in the CNS display properties of adult stem cells, responding to injuries and contributing substantially to other neural lineage cells, particularly scar-forming astrocytes (Johansson et al., 1999; Meletis et al., 2008; Carlén et al., 2009). These reports suggested that EPCs may also be a source of new remyelinating cells in CNS. However, our data argue against this prediction because, using a FGFR3 fate-mapping approach that labels EPCs as well as astrocytes, we could not detect any remyelinating SCs labeled in any demyelinating lesions in the spinal cord. Moreover, no pronounced proliferation of EPCs in the central canal at the level of focal demyelination lesions was detected. These results lead us to conclude that, despite their stem cell characteristics and involvement in other CNS injury models, EPCs in the spinal cord do not contribute to remyelination. The reasons for the discrepancy are not entirely clear. One plausible explanation could be that there is little or no direct EPC damage in focal demyelination model or in the experimental autoimmune encephalomyelitis model used by others (Guo et al., 2011), whereas this is likely to occur in the “stab” injury model used to provide evidence for a significant EPC role in regeneration (Barnabé-Heider et al., 2010). Other studies show that, whereas there is no significant change in EPC proliferation in central canal following pure demyelinating injury, this does occur following traumatic injury (Lacroix et al., 2014) and the involvement is dependent on direct ependymal injury (Ren et al., 2017). It is also possible that, because different fate-mapping mouse lines were used in this study and that by Guo et al. (2011), different subpopulations or additional population of cells are labeled.

We conclude that the Foxj1-labeled remyelinating SCs came from the PNS. Early studies on the presence of SCs in the demyelinated CNS, either in human diseases or animal models, suggested that these cells were likely derived from SCs that invaded the CNS from the PNS (Blakemore, 1975; Itoyama et al., 1983; Dusart et al., 1992; Duncan and Hoffman, 1997; Jasmin et al., 2000). This conclusion was largely based on the proximity of CNS SC remyelination to the spinal root transition zone (although SC remyelination has also been reported in CNS areas such as the cerebellar peduncles that are more remote from an obvious PNS source of SCs). However, our genetic fate-mapping experiments, which allowed tracing of prelabeled P0-expressing myelinating SCs, showed that the contribution of existing SCs to CNS remyelination was minimal and that most were derived from CNS-resident OPCs (Zawadzka et al., 2010). These findings led to the conclusion that nearly all remyelinating cells in the CNS lesions are of CNS origin. However, fate mapping of PNS cells using the P0-driven reporter lines did not exclude the possibility that other PNS cells including NMSCs might also contribute (because P0 is largely expressed by myelinating SCs) (Jessen and Mirsky, 2005). The current study with lineage tracing demonstrated that, following demyelination of the ventral spinal cord, many remyelinating SCs came from Foxj1-labeled cells from peripheral nerves. The extent of the peripheral contribution of CNS remyelination is location dependent, invariably occurring adjacent to the spinal root entry zone, where the glia limitans has likely been breached (Duncan and Hoffman, 1997). In areas relatively distant to peripheral nerves, where there is no obvious breach in the glia limitans, no Foxj1-GFP expression was evident even though there was substantial SC-mediated remyelination in the lesions, which is therefore likely to be OPC derived. Our data also suggest that proximity of demyelinating CNS lesions to spinal roots is likely necessary for Foxj1^+^ cells to contribute to CNS remyelination. In ventral spinal cord lesions, GFP^+^ cells could not be detected unless they were adjacent to a ventral root. Requirement of direct injury of spinal roots may also be necessary to mobilize the Foxj1^+^ cells because proliferating and remyelinating Foxj1-GFP labeled cells occur only in the ventral roots of lesion side, not on the contralateral side.

We have provided evidence that the Foxj1-expressing cells in peripheral nerves are a subpopulation of NMSCs. Both myelinating SCs and NMSCs are differentiated from SC precursors, which originate from neural crest stem cells (Jessen and Mirsky, 2005, 2016; Taveggia et al., 2005). In response to axonal injury, both myelinating SCs and NMSCs undergo a cellular reprogramming to assume an immature phenotype mediated by activation of transcription factor c-Jun to become “repair SCs” (Bungner cells) (Arthur-Farraj et al., 2012). Although repair SCs from the two origins are rarely distinguishable, subtle differences in how the two types of mature SCs respond to nerve injuries may exist. However, establishing whether this is the case has been hampered by markers that unambiguously distinguish the two types of mature cell. Grafting from sensory nerves containing NMSCs into injured motor nerves, which have few NMSCs, yields a “sensory pattern” in growth factor expression, suggesting that injury-activated repair SCs of NMSC origin may have different roles in regulating the regenerative response to injury (Höke et al., 2006). Our data support a distinctive regenerative capacity in cells derived from NMSC because it is these cells that seem predominantly able to contribute to CNS remyelination. Relevant to our study, it is clear that NMSCs do not need their own axon to be damaged, but instead are able to respond to injury-associated diffusible signals from damaged myelinated fibers, which elicit proliferation of NMSCs (Murinson and Griffin, 2004). It remains to be established what signals generated by CNS white matter demyelinating injury can activate and mobilize Foxj1 NMSCs in nearby peripheral nerves.

What is the nature of the Foxj1-expressing cells in peripheral nerves? We believe these cells to be NMSCs (based largely on the immunoelectron microscopy evidence). However, they also express markers more commonly associated with fibroblasts such as fibronectin, P4HB, and HSP47 (Parrinello et al., 2010) in intact peripheral nerves and *in vitro*. Endoneurial fibroblasts are derived from neural crest stem cells during development (Joseph et al., 2004) and play a role in SC sorting during peripheral nerve injury (Parrinello et al., 2010). However, their molecular identity seems unclear and they lack specific markers (Richard et al., 2012). The expression of NG2 and other markers in fibroblasts or NMSCs has been a subject of discussion that has not been completely resolved (Martin et al., 2001; Zhang et al., 2001; Rezajooi et al., 2004). The expression of fibroblast markers implies that their mesenchymal features allow their regenerative capacity, which is induced in dedifferentiated SCs following injury (Clements et al., 2017). In our studies, we were able to distinguish between a PDGFRA^+^ fibroblast, which does not contribute to generation of either CNS or PNS remyelinating SCs (Zawadzka et al., 2010; Assinck et al., 2017), and a Foxj1^+^ NMSC with fibroblast features, which is able to generate remyelinating SCs in both the CNS and PNS. It cannot be excluded that the L1^−^Foxj1^+^ cells may be a separate population from NMSCs and are not of PDGFRA^+^ fibroblast-like population in the PNS. The identification of this population is hampered by low efficiency in transgene induction by tamoxifen, promiscuity of fibroblast markers, and the limitations of immunohistochemistry. However, the boundary of these cell types may be naturally overlapping given that some of them have a shared developmental origin from neural crest progenitors. Another potential source of cells expressing similar markers is pericytes, but they are unlikely to overlap with the Foxj1-GFP population because our unpublished fate-mapping data showed that PDGFRb-GFP cells did not give rise to SCs following sciatic nerve crush. There is a population of cells called boundary cap cells identified only in developmental spinal roots at the boundary of CNS and PNS, which had been described to have stem cell properties (Zujovic et al., 2010). However, these cells are unlikely to be associated with Foxj1^+^ cells because they have never been found in the adult peripheral nerves and they behave differently by making remyelinating oligodendrocytes instead of SCs when grafted in CNS lesions (Zujovic et al., 2010, 2011).
